# Comparative Sequence Analysis of *TRI1* of *Fusarium*

**DOI:** 10.3390/toxins11120689

**Published:** 2019-11-23

**Authors:** Amanda C. Ramdass, Ria T. Villafana, Sephra N. Rampersad

**Affiliations:** Department of Life Sciences, Faculty of Science and Technology, The University of the West Indies, St. Augustine, Trinidad and Tobago; ac_ramdass@hotmail.com (A.C.R.); riatvill@hotmail.com (R.T.V.)

**Keywords:** cytochrome P450, *Fusarium*, *TRI1*, NX-2 trichothecene

## Abstract

Trichothecene mycotoxins are a class of secondary metabolites produced by multiple genera of fungi, including certain plant pathogenic *Fusarium* species. Functional variation in the *TRI1* gene produces a novel Type A trichothecene called NX-2 in strains of *F. graminearum*. Using a bioinformatics approach, a systematic analysis of 52 translated *TRI1* sequences of *Fusarium* species, including five *F. graminearum* NX-2 producers and four *F. graminearum* non-NX-2 producers, was conducted to explain the functional difference of *TRI1p* of FGNX-2. An assessment of several signature motifs of fungal P450s revealed amino acid substitutions in addition to the post-translational N-X-S/T sequons motif, which is indicative of N-linked glycosylation of this *TRI1*-encoded protein characteristic of NX-2 producers. There was evidence of selection bias, where *TRI1* gene sequences were found to be under positive selection and, therefore, under functional constraints. The cumulative amino acid changes in the *TRI1p* sequences were reflected in the phylogenetic analyses which revealed species-specific clustering with a distinct separation of FGNX-2 from FG-non-NX-2 producers with high bootstrap support. Together, our findings provide insight into the amino acid sequence features responsible for the functional diversification of this *TRI1p*.

## 1. Introduction

Trichothecenes mycotoxins are a large class of sesquiterpene metabolites, which are produced by several genera of plant pathogenic fungi including several species of *Fusarium*. Trichothecenes are considered to be virulence factors and their production improves the pathogenic potential of *Fusarium* species in a number of economically important plant host species [1,2,3,4]. Trichothecenes can be classified into four types based on (i) the carbonyl group at the 8-position, (ii) the position of macrolide rings, and (iii) the number of epoxy rings [5]. Type A (e.g., T-2 toxin and HT-2 toxin) and Type B trichothecenes (e.g., deoxynivalenol (DON) and nivalenol (NIV)) are harmful after acute and chronic exposure, highly cytotoxic, pro-inflammatory with emetogenic properties and are efficient inhibitors of eukaryotic protein synthesis [6,7,8]. The Food and Drug Administration (FDA) in the USA and the European Commission, Europa EU, enforce maximum allowable limits of trichothecene contamination of food products used in national and international trade (International Trade and Food Safety/AER-828, Economic Research Service/USDA; https://www.ers.usda.gov/webdocs/publications/41603/15640_aer828h_1_.pdf?v=42055).

**Figure 1:**
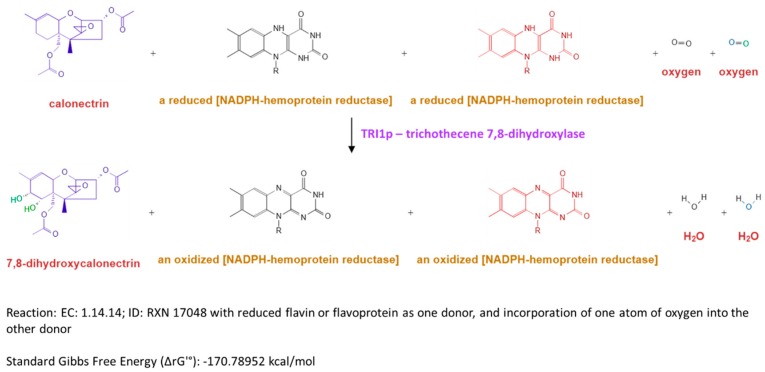
Hydroxylation reactions catalysed by trichothecene 7,8-dihydroxylase (MetaCyc Accession No. G-44257 [13] and UniProt Accession No. Q7Z886 (https://www.uniprot.org/uniprot/Q7Z886).

Biosynthesis of trichothecenes is carried out by expression of a core *TRI* gene cluster [4]. Depending on the *Fusarium* species, oxygenase (*TRI1*) and acyltransferase (*TRI16*) genes in addition to an acetyltransferase gene (*TRI101*) can be located external to the core [9]. The *TRI1* gene of different *Fusarium* species encode calonectrin oxygenase (*TRI1p*) [9] and its expression is regulated by transcription factors *TRI6p* and *TRI10p* [5,10]. Specifically, for *F. sporotrichioides*, the *TRI1* gene (*FsTRI1*) encodes a cytochrome P450 monooxygenase that hydroxylates C-8 of calonectrin in the biosynthesis of Type A trichothecenes. For *F. graminearum*, a homologue of this gene (*FgTRI1*) encodes a cytochrome P450 dioxygenase, trichothecene 7,8-dihydroxylase, that catalyzes the hydroxylation of both C-7 and C-8 of calonectrin to generate 7,8-dihydroxycalonectrin and/or generate the 7- and 8-monohydroxylated calonectrin in the biosynthesis of DON and NIV [10,11,12,13] (Figure 1).

Functional analyses of *TRI* genes of the *TRI* biosynthetic gene cluster explained, in part, the basis for the structural diversity of trichothecene analogs produced by *Fusarium* species [4,9,12,14]. Proctor et al. [4] reported that *TRI* gene gain, loss, and subsequent alterations in function of these genes are the primary effectors of this structural diversity. As a result, chemotype shifts can occur as a result of the introduction of new genotypes into new areas [15]; for example, detection of 3-ADON genotypes of *F. graminearum* sensu stricto in Canada [16], 3-ADON genotypes of *F. asiaticum* in Southern China [17], and the NIV genotype of *F. asiaticum* in the southern parts of the USA [18] and Brazil [19].

The value of studying *TRI* genes has been demonstrated by the detection of a novel type A trichothecene toxin (NX-2) produced by specific strains of *F. graminearum* (FGNX-2) [20,21]. NX-2 and 3-ADON producing strains primarily generate the 3-acetylated derivative of trichothecenes, which are less toxic than their related metabolites [22]. Structural analysis of the NX-2 toxin indicated that a distinct variant of the *TRI1* gene product (*TRI1p*) catalyzes C-7 hydroxylation, but not C-8 hydroxylation of calonectrin [23]. This lack of a C-8 carbonyl group allows NX-2 toxin to escape detection by HPLC-UV-based methods. While this underlines the value of genotyping at this initial stage of investigation, it also demonstrates the value of LC-MS/MS or GC-MS methods of toxin detection. Initially, a low frequency of occurrence of NX-2 in northern US and in Canada was reported [20,21]. However, Lofgren et al. [24] more recently analyzed a large collection of *F. graminearum* strains from New York in the USA, and found that (i) the frequency of NX-2 genotype strains was up to 14 times higher than previously reported, (ii) NX-2 genotypes were detected in maize ears and stubble in addition to wheat heads, and (iii) 20% of the total *F. graminearum* population in the USA could be attributed to the NX-2 genotype. The detection of NX-2-producing strains in Canada, albeit at low frequency, reinforces the need for continued monitoring of *Fusarium* populations in wheat-growing regions of North America [21].

We present a study of 52 *TRI1* peptide sequences (*TRI1p*) of *Fusarium* species including five *F. graminearum* (as NX-2 producers, FGNX-2) and four *F. graminearum* (as non-NX-2 producers, FG- non-NX-2), in which systematic analysis of signature motifs of P450s and of protein sequence phylogeny are carried out in order to explain the functional difference of *TRI1p* of FGNX-2.

## 2. Results and Discussion

Note: In the figures, the five FGNX-2 and four FG-non-NX-2 sequences with 21 additional *Fusarium* sequences are shown for comparison, as the entire 52 *TRI1p* sequence dataset could not be depicted in any one figure.

### 2.1. TRI1 Peptide Primary Sequence Comparisons

**Figure 2:**
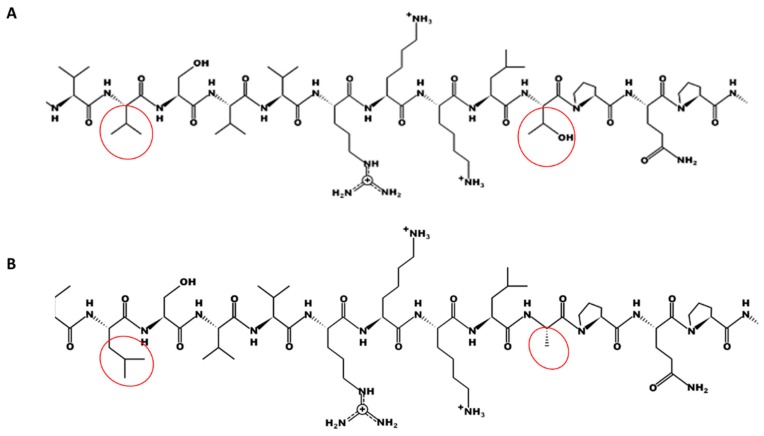
Comparison of primary structure *TRI1* peptide sequences (*TRI1p*) showing the specific sequence length where differences in the structure are apparent; **A**: Representative FGNX-2 isolate (GenBank Accession No. AIU41071); **B**: Representative FG-non-NX-2 isolate (GenBank Accession No. AOC89125). Red circle indicates specific differences between the primary sequences of FGNX-2 and of FG-non-NX-2.

**Figure 3:**
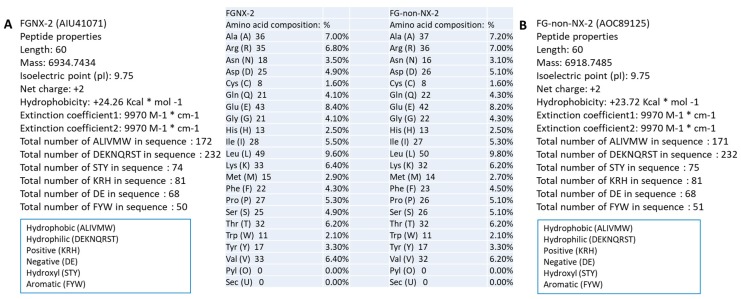
Comparison of *TRI1* peptide sequences (*TRI1p*) showing differences in amino acid composition; **A**: Representative FGNX-2 isolate (GenBank Accession No. AIU41071); **B**: Representative FG-non-NX-2 isolate (GenBank Accession No. AOC89125).

Comparison of the *TRI1p* sequences revealed differences in the amino acid composition, in the primary predicted peptide structure, and in chemical characteristics, e.g., the hydrophobicity was higher for FGNX-2 and the number of hydroxyl groups was higher for FG-non-NX-2 (Figure 2 and Figure 3A,B). A representative sequence of FGNX-2 (GenBank Accession No. AIU41071) and of FG-non-NX-2 (GenBank Accession No. AOC89125) are shown.

### 2.2. Topology

**Figure 4:**
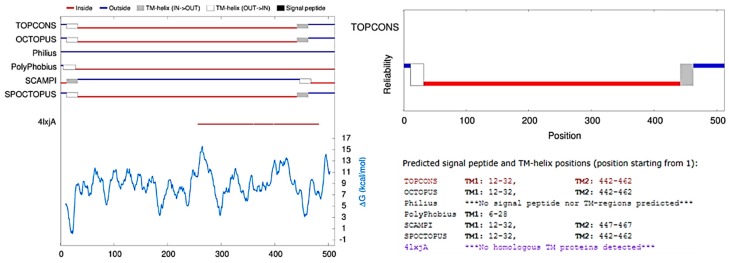
TOPCONS predicted topologies and *Δ*G values for FGNX-2 and FG-non-NX-2 *TRI1p* sequences.

Topology analysis indicated two transmembrane domains at the N- and C-terminus for all *TRI1p* sequences in five out of six prediction programs in TOPCONS2 (Figure 4). This finding is consistent with the data presented by Menke et al. [25], Kistler and Broz [26], and Boenisch et al. [27], in which trichothecene reaction products and intermediates are sub-cellularly compartmentalized and associated with the endoplasmic reticulum. Upon mycotoxin induction, the ER undergoes ultrastructural re-organization into proliferations of the organized smooth endoplasmic reticulum (SER) and *TRI1p* is associated with this modified ER membrane [27].

### 2.3. PER/PxRW and ExxR Motifs

**Figure 5:**
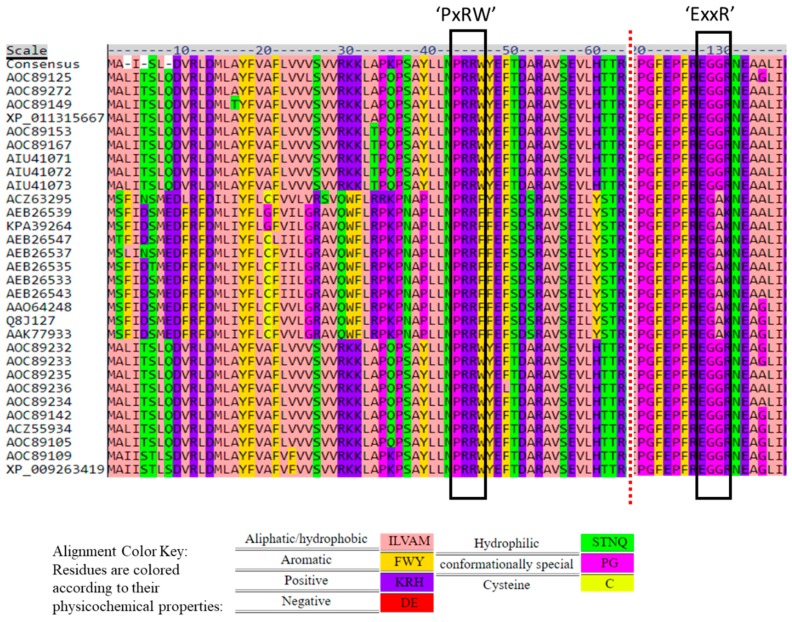
Signature ‘PxRW’ and ‘ExxR’ motifs in the *TRI1p* sequence of *Fusarium* species.

The alignment also revealed the conserved PER domain of P450s as the characteristic signature for fungi (PxRW) [28,29] (Figure 5). Two clusters were represented by PPRF while all other *Fusarium* species had PRRW. Both FGNX-2 and FG-non-NX-2 isolates had the PRRW motif.

Two clusters contained variations of this ExxR motif as Eggf and Eggm, while other *Fusarium* clusters contained EggR. The ExxR and PER motifs form “the E-R-R triad”, which functions to secure the heme pocket into its correct position, and thus, to ensure stabilization of the core structure of the enzyme [30]. Both FGNX-2 and non-NX-2 producers had the EggR motif (Figure 5). Sello et al. [31] analyzed the ExxR motif among oomycetes and found that the first and fourth residues, i.e., “E” and “R” are conserved in all P450 families, e.g., CYP5014, CYP5015, and CYP5017. There are a few exceptions; in CYP5017F8, the motif consisted of a “K” instead of “E” and in CYP5014N1 and CYP5015L, the motif consisted of “W” and “H”, respectively, instead of “R”. Variations of the “E” and “R” amino acids at the ExxR motif are uncommon [32].

### 2.4. Dileucine (LL) Motifs

**Figure 6:**
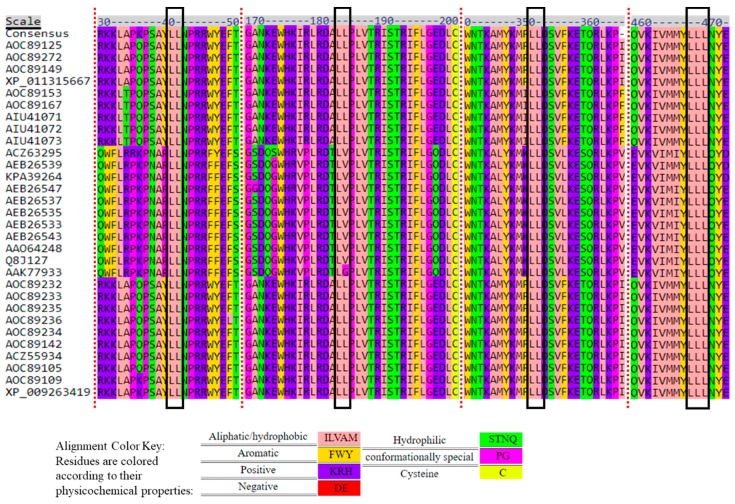
Dileucine repeats in the *TRI1p* sequence of *Fusarium* species.

WoLFPSORT detected four dileucine sequence motifs (LL) based on the alignment of 52 *TRI1p* sequences (Figure 6). The function of the LL sequence motif depends on the nature of the adjacent residues; however, there is no specific peptide sequence within which LL motif resides [33]. Several transmembrane proteins consist of LL motifs that function as sorting signals [34]. Although dileucine sequence motifs are not necessarily characteristic of P450s, their detection was still included here.

### 2.5. Heme Motif

P450s have a signature heme motif sequence FXXGX_b_XXCXG, where X_b_ is a basic amino acid and the cysteine residue serves as the catalytic ligand located axial to the heme moiety, i.e., the specific thiolate group of the cysteine amino acid structure occupies the axial coordination site of iron opposite to the bound oxygen [35,36]. Substrates, due to their hydrophobicity, bind in a cleft or pocket above heme.

**Figure 7:**
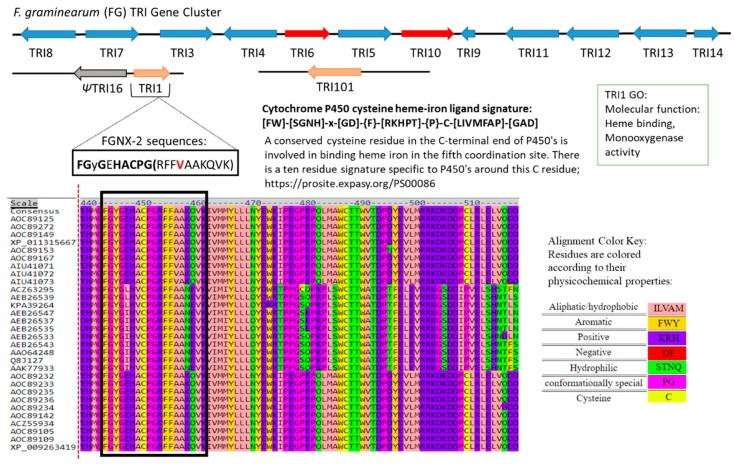
Variation in heme motif of *TRI1p* amino acid sequences of the *Fusarium* species.

**Figure 8:**
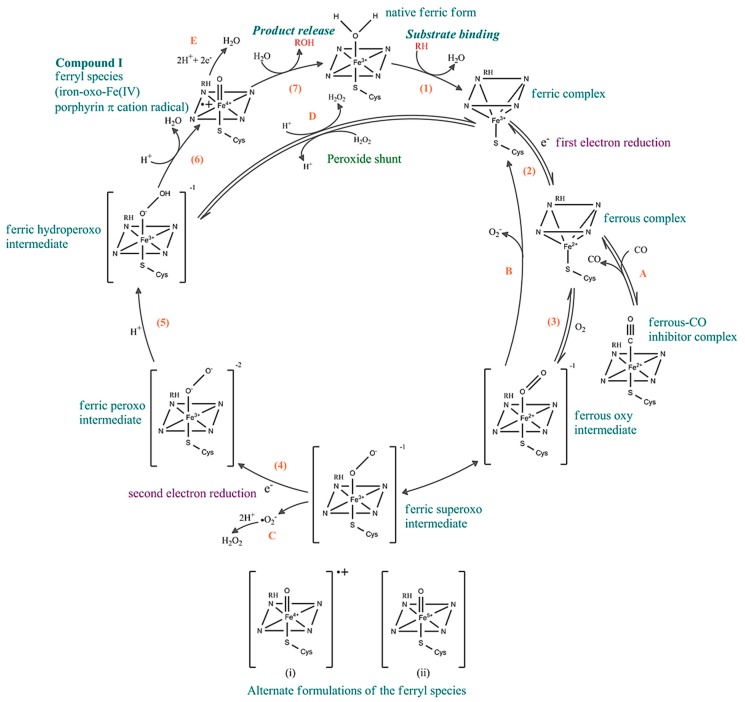
Catalytic cycle of cytochrome P450. The heme macrocycle is depicted to represent the resting enzyme which is in the ferric state and has a thiolate proximal ligand (cysteine thiolate, indicated as an S-Cys atom linked to the iron) and a distal ligand (a water molecule, which changes to dioxygen as the cycle proceeds). (1) The substrate (RH) binds in proximity to the heme group displacing the distal water ligand leading to a conformational change in the active site of the P450 molecule and a shift in the ferric heme iron spin-state equilibrium from low-spin (S = 1/2) to a high-spin (S = 5/2). (2) This leads to a shift in the redox potential that enables electron transfer from a redox partner to occur to reduce the heme iron to the ferrous state. The first electron transfer occurs which reduces the ferric iron (heme-Fe(III)) to its ferrous form (heme-Fe(II)). The ferric complex in the presence H_2_O_2_ or any organic peroxy compound (e.g., an alkyl hydroperoxide or peracid) can lead to the production of the ferric hydroperoxo intermediate (although inefficiently in most cases). (3) Oxygen then binds to the ferrous heme center to produce the ferrous-oxy intermediate that is isoelectronic with the ferric superoxy form. (4) A second electron transfer occurs reducing the heme iron to the ferric peroxo intermediate. This electron transfer step is usually, but not always, the rate-limiting step in the cytochrome P450 catalytic cycle. (5) Rapid protonation of the peroxo intermediate leads to the ferric hydroperoxo intermediate. (6) The hydroperoxo formed in step (5) is unstable and undergoes rapid protonation leading to scission of the dioxygen bond with the production of a water molecule and the generation of iron-oxo-Fe(IV) porphyrin π cation radical (ferryl species) also known as Compound I. This is considered to be the catalytically reactive substrate oxidant in most cytochrome P450 reactions. Alternative formulations of the ferryl intermediate shown are as (i) a protein radical cation Fe(IV) species or (ii) as an Fe(V) species. (7) Attack of the nearby substrate by the ferryl species effects its hydroxylation and metabolite/product (ROH) dissociation from the cytochrome P450 molecule via oxygen insertion from the ferryl species into the substrate. Product dissociation allows water to rebind to the ferric iron and complete the cycle. Within the cycle non-productive pathways leading to the collapse of intermediates are also indicated. (A) Irreversible denaturation to an inactive form of the cytochrome P450 ferrous complex yields a ferrous-CO complex. (B) Decay of the ferrous oxy intermediate leads to the reformation of ferric P450 complex with the production of superoxide. (C) Reduction of molecular oxygen in this step also results in the release of •O_2_ and H_2_O_2_ due to the decay of the ferric superoxo intermediate. (D) In the peroxide shunt the ferric hydroperoxo species can collapse with the release of peroxide. (E) The decay/collapse of the ferryl species via the addition of two electrons results in the release of water. Factors such as untimely electron/proton delivery or if the substrate is inappropriately positioned or resistant to oxidative attack can result in the collapse of this species [37,38,39,40,41].

Analysis of the 52-amino acid sequence alignment revealed that FGNX-2 producers have a variant amino acid sequence signature for heme compared to the 10-amino acid consensus sequence: [FW]-[SGNH]-x-[GD]-{F}-[RKHPT]-{P}-C-[LIVMFAP]-[GAD], where C is cysteine residue that interacts with the heme iron ligand (Figure 7; Figure 8). This motif is located at the C-terminus of the P450 and this finding is supported by Menke et al. [25], who reported that the heme binding domains, and, therefore, the enzyme active sites are near the C-terminus.

Varga et al. [22] analyzed amino acid sequences of *F. graminearum* DON-producers and *F. graminearum* NX-2 producers and found that NX-2 producers had a distinct heme binding motif according to a 20 amino acid heme binding motif. Sello et al. [31] analyzed the heme motif across three P450 families and reported that amino acid residues “F”, “G” and “C” located as the first, fourth, and eighth positions in the heme motif are conserved in among P450s across biological kingdoms [32], but there are some P450s with different amino acids at these positions [42].

**Figure 9:**
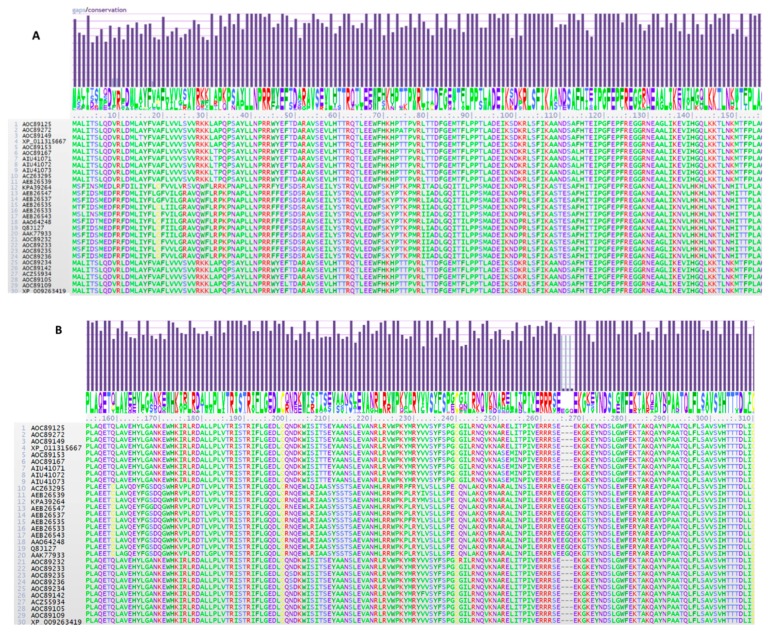

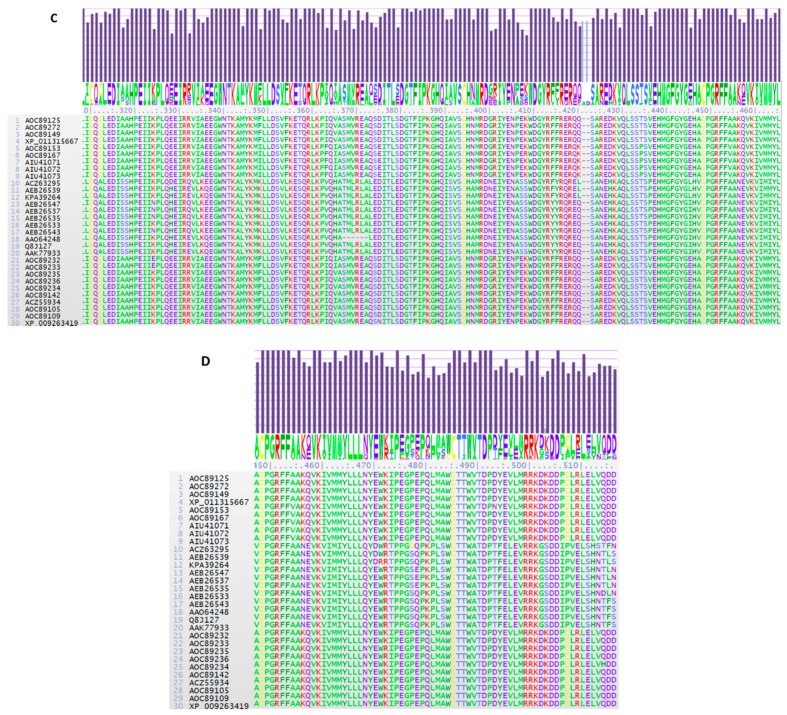
**A**–**D**. Single amino acid substitutions of 30 representative *TRI1* peptide sequences including those of FG-non-NX-2 isolates (numbers 1 to 4) and of FGNX-2 (numbers 5 to 9); 21 sequences belonged to the other *Fusarium* species; the entire 52-sequence alignment could not be shown here.

P450-catalyzed hydroxylation is the most characteristic reaction catalyzed by P450 enzymes (Figure 9); however, substrate specificity is determined by three factors.

(i) Substrate lipophilicity affects its compatibility with the P450 active site architecture and ultimately influences the affinity of the substrate for the P450 active site. The domain located above the heme group is relatively hydrophobic compared to other enzymes and interactions with substrates are driven entirely by lipophilic contacts [43].

(ii) In a case where oxidation of C-H bond is controlled by intrinsic reactivity rather than by steric constraints or positioning of the substrate within the active site, the C-H bond strength determines the reactivity of the substrate [43].

(iii) The size and shape of P450 active sites impose substrate selectivity [30]. Substrates are situated in catalytic pockets where the atom to be hydroxylated is oriented within a specific distance from the heme iron depending on the P450 with restricted mobility [43]. Regio- and stereo-selective hydroxylation is enabled by specific active site-substrate interactions that position the substrate for oxidation [36]. P450s with high catalytic specificity have key residues involved in orientation and steric interactions between the substrate and the protein residues of the active site, and it is these imposed steric barriers that affect access of the ferryl species [44]. Regio-selectivity is mainly controlled by amino acid residues at the active site of P450s and specific amino acid substitutions within the active site have the potential to interact with an aromatic hydrocarbon substrate to induce selective hydroxylation of the ortho- or meta- or para-position despite having a native preference for one or the other [45]. What controls aromatic oxidation is that position that is electron rich and has the least steric hindrance by other substituents; +R substituents give product mixtures that have relatively high combined yields of ortho- and para- products [46]. With respect to oxidation of mono-substituted benzene rings, ortho- para-directors include hydroxyl groups [47,48]. This may also explain why hydroxylation of calonectrin at C-7 (para-position) is preferred over C-8 hydroxylation (meta-position) in *TRI1p* of FGNX-2. Unique combinations of amino acid patterns detected at ExxR and heme motifs suggest that these variant motifs are perhaps characteristic of a particular P450 subfamily.

Supplementary Figures S1 and S2 show the details of substrate binding relative to the heme moiety.

### 2.6. TRI1 DNA Polymorphism Profile and Evidence of Selection

Silent mutations are not manifested in an organism’s phenotype. Silent mutations in the nucleotide sequence of the *TRI1* gene will not result in an amino acid change and there should be no alteration of enzyme function. When FGNX-2 *TRI1* nucleotide sequences (*N* = 5) were compared with FG-non-NX-2 (*N* = 4) in DnaSP 6, there were minor differences in DNA polymorphism profiles between the two datasets (Table 1). This is in contrast to DNA polymorphism analysis of the dataset consisting of 52 nucleotide sequences of all *Fusarium* species which indicated that the *TRI1* gene is highly polymorphic with the exception of significant nucleotide sequence conservation at nucleotide positions nt 1–36 [ATGGCTTTGATTACTTCATTGCAAGATGTTAGATTG] (*p* = 0.0013).

Conversely, synonymous mutations result in changes to the amino acid sequence that can affect transcription, post-transcriptional modifications, mRNA export, and translation, and which result in alterations to the structure and function of the protein. Analysis of 52 *TRI1* nucleotide sequences in DnaSP 6 revealed that the ratio of non-synonymous to synonymous substitutions (d*N*/d*S* ratio) was 3.54, which is >1. Fu and Li’s D* statistic was also positive and significant (2.05433; *p* < 0.02), and thus, it is inferred that *TRI1* gene sequences are under positive selection and under selective functional constraints. However, Kelly et al. [49] reported that tests of positive selection were not significant and the *TRI1* gene sequences of the FGNX-2 were identical except for one nucleotide. In a subsequent *F. graminearum* genome study by Kelly and Ward [50], it was reported that among several genomic regions, *TRI* genes exhibit the strongest signals of selection. These non-synonymous substitutions that are restricted to FGNX-2 *TRI1* sequences suggest that the emergence of the NX-2 genotype may have been driven by changes in selection pressure on this gene [49] and perhaps this genotype is a transient P450 conformation [51].

### 2.7. TRI1p Residue Substitutions

*TRI1p* sequences of the five FGNX-2 isolates were highly conserved. When compared with other *Fusarium* species, however, there were approximately six amino acid substitutions specific only to FGNX-2 sequences that contributed to distinct resolution of FGNX-2 sequences from all other *Fusarium TRI1p* sequences (Figure 9A–D). Amino acid substitutions specific to FGNX-2 sequences were: at alignment position 252 A/R/N>S; at alignment position 254 L>M; at alignment position 349 F/K>I; at alignment position 421 Q/E/L>K; at alignment position 435 T>P; at alignment position 455 A > V. Kelly et al. [49] reported that there were no differences in the predicted amino acid sequences of FGNX-2 but, detected 14 amino acid differences specifically between the *TRI1* gene product of FGNX-2 strains and *F. graminearum* strain PH-1 (non-NX-2-producer) due analysis of a larger dataset.

### 2.8. Phylogeny of TRI1p Sequences of Fusarium

**Figure 10:**
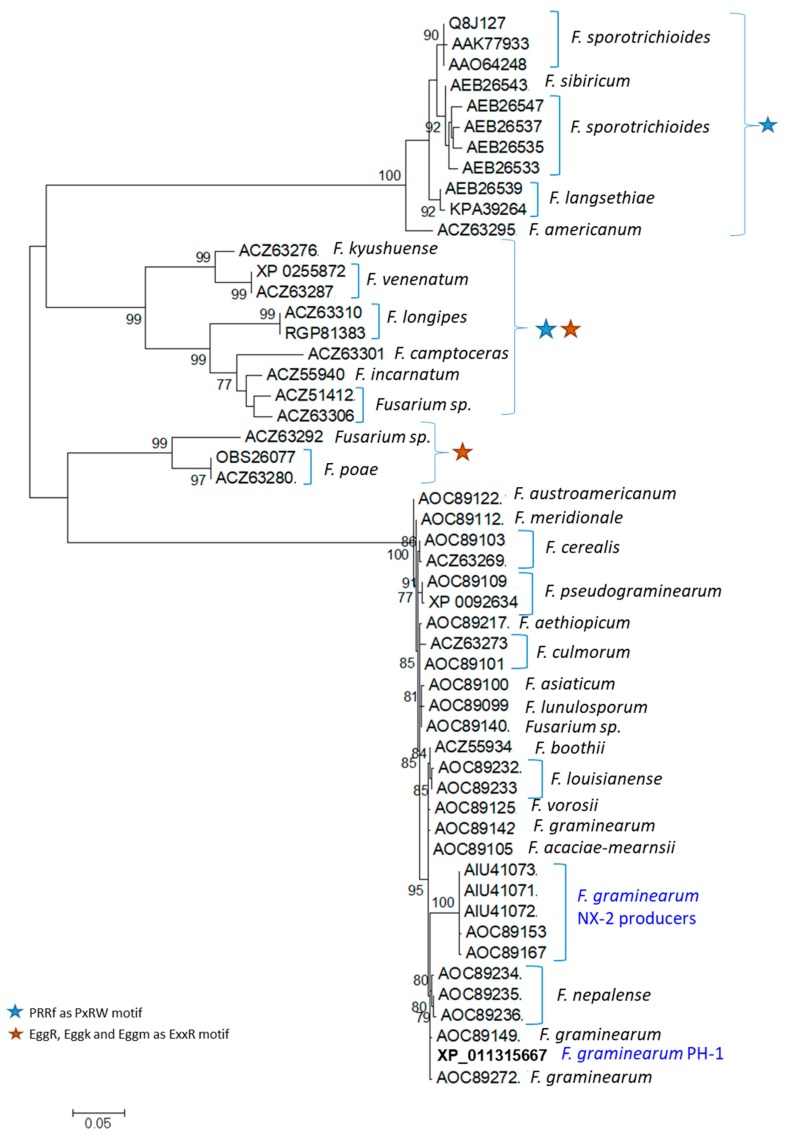
Maximum likelihood phylogenetic tree based on an alignment of 52 amino acid sequences of *TRI1p* of *Fusarium* species.

This difference in *TRI1p* amino acid substitution among *Fusarium* species is also shown in the phylogenetic tree produced in RaXML (Figure 10). Species-specific clustering was indicated with moderate to high bootstrap support (>75% and >90%, respectively) for most taxa. It was also apparent that residue substitutions in the PxRW and ExxR motifs accumulated in three clusters of *Fusarium* species which excluded *F. graminearum TRI1p* sequences. In addition, there was also distinct separation of FGNX-2 from FG-non-NX-2 isolates with high bootstrap support (>99%).

### 2.9. N-Linked Glycosylation

**Figure 11:**
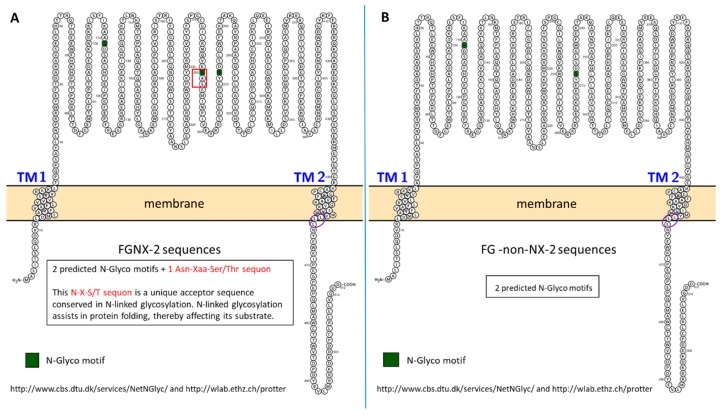
Comparative NetNGlyc prediction of N-linked glycosylation of asparagine amino acid in the *TRI1p* sequence of FGNX-2 and FG-non-NX-2 isolates. **A**: Representative FGNX-2 sequence, **B**: Representative FG-non-NX-2 sequence. Red frame indicates the location of a N-X-S/T sequon found only in FGNX-2 sequences. Purple circle indicates common location of dileucine repeat in both FGNX-2 and FG-non-NX-2 sequences.

Based on NetNGlyc (http://www.cbs.dtu.dk/services/NetNGlyc/) predictions, only the *TRI1p* sequences of FGNX-2-producers have a definite N-X-S/T sequon motif; this motif is absent in non-NX-2-producers (Figure 11). This N-X-S/T sequon is conserved in N-linked glycosylation. N-linked glycosylation is a critical post-translational modification of proteins that are synthesized and folded in the endoplasmic reticulum [52].

There are >900 P450 structures available in the Protein Data Bank (http://www.wwpdb.org/), many of which contain bound ligands. Structural flexibility enables an expansion of the substrate spectrum of a P450 due to flexibility of the active site where substrate docking occurs. Most P450s have a conserved basic P450 structural fold, but their substrate-binding pockets demonstrate high structural plasticity which enables these enzymes to significantly vary the dimensions of their active sites according to the chemical structure of the substrates and this drives catalytic selectivity [36,43]. Thus, the existence of multiple conformations of P450s is reflected in multiple docking models [53]. It follows that a given substrate can bind in multiple orientations depending on regio- and/or stereo-selectivity of the active site. In extreme cases, a single amino acid substitution may be enough to change an enzyme’s regio-specificity and catalytic efficiency [54]. *TRI1p* can apparently utilize calonectrin, 3, 15-DAS and, to a lesser extent, isotrichodermin as substrates in the production of trichothecene mycotoxins, which suggests that the *TRI1p* active site has a relaxed substrate specificity [14,55].

In view of the role of the heme-Fe complex and the conformational interactions of specific amino acid residues in P450-driven oxidation, it is hypothesized that the *TRI1p* variant heme sequence and select amino acid substitutions of NX-2-producers may affect regio- and stero-selectivity for substrate (calonectrin) docking, orientation and position for oxidation relative to the heme-Fe complex. Substitution of key residues would affect the ability of the substrate to make multiple orientations in the active site relative to the heme-Fe complex. Furthermore, the N-X-S/T sequon, which is tagged for N-linked glycosylation that appears to be unique to FGNX-2, would impact upon folding of the protein compared to non-NX-2- producers for which this sequon is absent. It is an advantage to P450 chemistry if the correct tertiary structure is retained as P450s also repositions their active site residues upon substrate binding [51].

According to Kimura et al. [14], closely-related *Fusarium* species (i) produce trichothecenes as different structural variants due to substitution patterns of functional groups at C-3, C-4, C-7, C-8, and C-15, (ii) the late stages of trichothecene biosynthesis differs for Type A and Type B trichothecene production as a result of substrate specificity, and (iii) trichothecene biosynthesis operates along “metabolic grids” rather than linear pathways. As such, 7-hydroxycalonectrin and 8- hydroxycalonectrin can both be used as substrates for generation of DON. Although there is no specific data on the ratio of 7, hydroxycalonectrin to 8, hydroxycalonectrin produced, their production is sequential and not random [14,56]. Furthermore, structural diversity that results in altered *TRI* gene function (acetylation, acylation, and hydroxylation) in different fungal genera that produce these mycotoxins and the current *TRI* gene functions can be either ancestral, derived, or demonstrate retained, but attenuated ancestral gene function [4].

Although the folded structure and catalytic competence of P450s must be maintained, the active sites can tolerate certain mutations and still retain its function. It is perhaps this mutational robustness that reflects the diversity of the P450 family, the key role of the iron-heme prosthetic group in catalysis, the hydrophobicity of the active site, and the conformational variability upon substrate binding [51]. Although most random mutations are either neutral or deleterious, mutation fixation in certain *F. graminearum* genomes indicates that these heme motif mutations may confer an adaptive advantage that is not yet defined [57,58,59,60].

## 3. Conclusions

*TRI1* gene sequences are more divergent among different *Fusarium* species than among closely related species [14]. It is important to understand the molecular mechanisms that drive the different substitution patterns at the later stages of trichothecene biosynthesis because the relative toxicity of trichothecenes is determined by the pattern of oxygenation, acetylation, and/or esterification of different substrates [14]. Furthermore, if a significant change in selective pressure enabled motif and amino acid variation in NX-2-producers, monitoring of the NX-2 genotype according to host and geographical range in an effort to identify potential source and range expansions should be carried out [49]. It is also relevant to understand whether the NX-2 genotype confers a selective advantage over other *TRI* genotypes [60].

## 4. Materials and Methods

**Figure 12:**
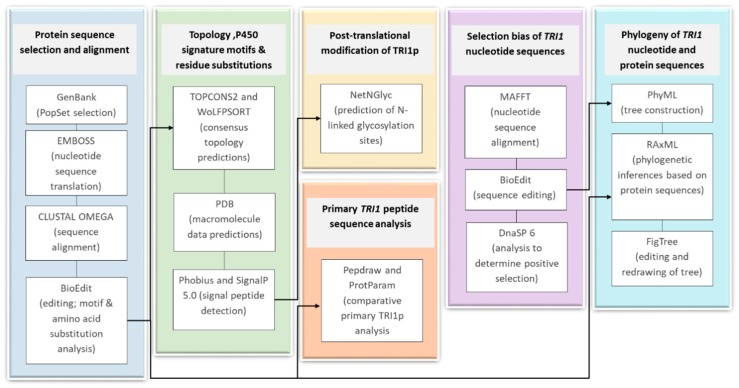
Bioinformatics pipeline developed for analysis of *TRI1* nucleotide and protein sequences.

The bioinformatics pipeline developed for analysis of *TRI1* nucleotide and protein sequences are outlined in Figure 12.

### 4.1. Protein Sequence Selection and Alignment

*TRI1* nucleotide sequences of different *Fusarium* species were accessed as PopSets in GenBank: PopSet: 699128280 [20] and PopSet: 1052473830 [49]. These sequences were selected, as they included *F. graminearum* NX-2 producers from two different studies [20,49], other *F. graminearum* strains as a non-NX-2 producers as well as *TRI1* sequences of other *Fusarium* species to be used as additional references. The sequence identities of all 52 sequences were verified in NCBI Multiple Sequence Alignment Viewer (MSAViewer: https://www.ncbi.nlm.nih.gov/projects/msaviewer/). Nucleotide sequences were translated (https://www.ebi.ac.uk/Tools/st/emboss_transeq/) and the amino acid sequences were aligned in CLUSTAL OMEGA (https://www.ebi.ac.uk/Tools/msa/clustalo/) and trimmed to a common length and analyzed for motifs and amino acid substitutions in BioEdit.

### 4.2. Comparative Primary Peptide Sequence Analysis

Primary sequence structure of the *TRI1* peptide of FGNX-2 and FG-non-NX-2 sequences were predicted and drawn on pepdraw (http://pepdraw.com/). The FGNX-2 protein sequences are AOC89153, AIU41071, AIU41072, AIU41073, and AOC89167. The FG-non-NX-2 protein sequences are AOC89125, AOC89149, AOC89272, and XP_011315667. The proteins’ theoretical properties were also calculated in NPS-Network Protein Sequence Analysis (https://npsa-prabi.ibcp.fr/) and ProtParam-Protein Identification and Analysis Tools on the ExPASy Server (https://web.expasy.org/protparam/) [61].

Alignment of *TRI1p* of FGNX-2 and a representative FG-non-NX-2 was compared in the Sequence Manipulation Suite (https://www.bioinformatics.org/sms2/color_align_prop.html). The color represents the biochemical properties of a particular residue.

### 4.3. Topology Analysis

Topology analysis was carried out in TOPCONS2-Consensus prediction of membrane protein topology and signal peptides (http://topcons.cbr.su.se/pred/result/rst_gW7Qq0/prediction) [62] and in WoLFPSORT (https://wolfpsort.hgc.jp/). Topologies for FGNX-2 and a representative FG-non-NX-2 were based on structure prediction and modeling against macromolecule data in the Protein Data Bank (PDB). Phobius (http://phobius.sbc.su.se/; http://phobius.sbc.su.se/poly) [63] and SignalP 5.0 (http://www.cbs.dtu.dk/services/SignalP/) [64] were used to determine presence/absence and location(s) of signal peptide sequences.

### 4.4. Signature Motifs of P450s

The alignment of 52 *TRI1p* sequences was examined for characteristic signature motifs of fungal P450s as identified by Syed and Mashele [42]. These motifs have been identified as conserved among P450 tertiary structure and enzyme functions [30]. Therefore, our specific objective was to detect *TRI1p* motif differences between FGNX-2 producers and non-NX-2 producers.

### 4.5. Selection Bias

Alignment of nucleotide sequences was carried out in MAFFT (Multiple Alignment using Fast Fourier Transform) server (https://www.ebi.ac.uk/Tools/msa/mafft/) and aligned sequences were then edited to a common length prior to analysis. DnaSP 6 [65] was used out to determine if *TRI1* gene sequences were under positive selection by assessing the ratio of non-synonymous to synonymous substitutions (d*N*/d*S*) [66] by using Fu and Li’s D* test statistic at *p* < 0.02 [67,68].

### 4.6. Phylogeny of TRI1 Nucleotide and Protein Sequences

Inferred *TRI1* phylogenetic relationships among *Fusarium* species were analyzed using the Maximum Likelihood (ML) algorithm. Phylogenetic inference of *TRI1p* sequences was estimated in RAxML [69] v0.9.0 (https://raxml-ng.vital-it.ch) using the maximum likelihood optimality criterion. The 75% consensus trees were retained and re-drawn in FigTree (http://tree.bio.ed.ac.uk/software/figtree/). Clusters for which motif variation were detected are also indicated as blue- and orange-colored icons on the tree.

### 4.7. Post-Translational Modification of TRI1p

NetNGlyc (http://www.cbs.dtu.dk/services/NetNGlyc/) predicts the number and location of N-Glycosylation sites using artificial neural networks that examine the sequence context of Asn-Xaa-Ser/Thr sequons (where Xaa is not proline). GPMAW lite (https://alphalyse.com/gpmaw/) was also used to detect N-glycosylation sites in the protein sequences.

## Figures and Tables

**Table 1:** Comparative DNA polymorphism profile for FGNX-2 and FG-non-NX-2 isolates based on an alignment of 1533 nucleotides of the partial *TRI1* gene.

DNA Polymorphism Parameter	FGNX-2	FG-Non-NX-2	52 *Fusarium* Sequences
*N*	5	4	52
*h*	3	2	46
H*d*	0.70000	1.00000	0.99500
*Pi*	0.00052	0.00065	0.30227
*k*	0.80000	1.00000	463.382
*Ct*	0.14	0.14	0.14
*C*	0.049	0.049	0.049

*N*—number of sequences in dataset; *h*—number of haplotypes; H*d*—haplotype differences; *Pi*—nucleotide diversity; *k*—nucleotide differences; C*t*—conservation threshold; C—sequence conservation.

## Supplementary Materials

The following are available online at https://www.mdpi.com/2072-6651/11/12/689/s1, Figure S1: Examples of heme interactions in a fungal cytochrome P450 monooxygenase, Figure S2: Example of substrate/ligand binding relative to heme moiety.
