# Supplementary material for: Comparative Sequence Analysis of TRI1 of Fusarium
Source: Toxins (Basel). 2019 Nov 23;11(12):689. doi: 10.3390/toxins11120689 (PMC6950058; doi:10.3390/toxins11120689)
Supplement: Supplementary file 1 [file toxins-11-00689-s001.pdf]

Supplementary Materials: Comparative Sequence Analysis of TRI1 of Fusarium

Amanda C. Ramdass, Ria T. Villafana and Sephra N. Rampersad

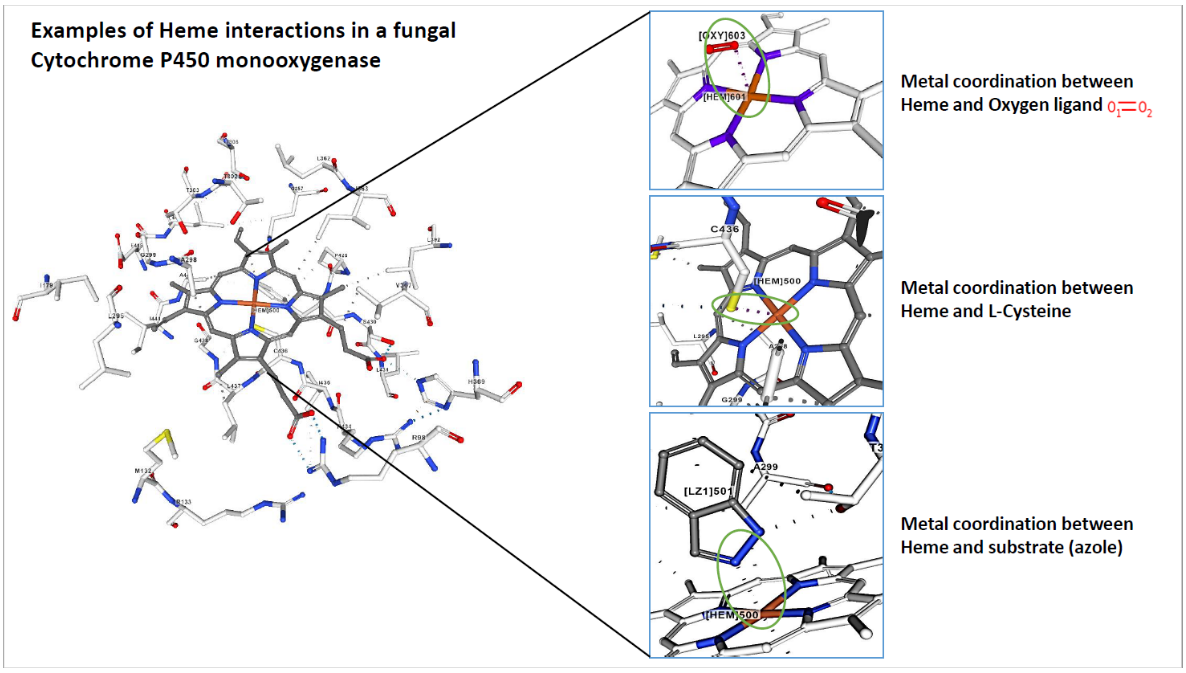

Figure S1. Examples of heme interactions in a fungal cytochrome P450 monooxygenase.

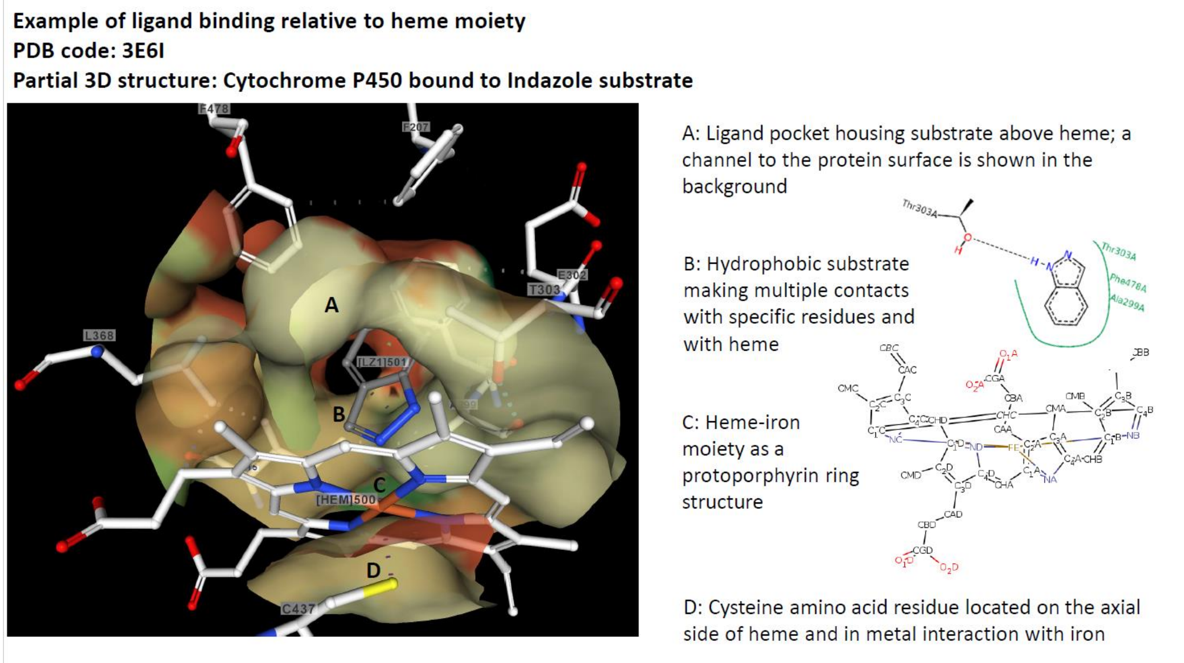

Figure S2. Example of substrate/ligand binding relative to heme moiety.
